# Hope into Achievement: A Longitudinal Examination of Hope, Psychosocial Perceptions, and Academic Achievement in a Sample of High School Students

**DOI:** 10.3390/bs15121657

**Published:** 2025-12-02

**Authors:** Dante D. Dixson, Ersie-Anastasia Gentzis, Leah Jansen, Kayla Whitley

**Affiliations:** 1 Department of Counseling, Educational Psychology, and Special Education, Michigan State University, East Lansing, MI 48824, USA; jansenl@msu.edu (L.J.); whitle25@msu.edu (K.W.); 2 Department of Counseling and Educational Psychology, New Mexico State University, Las Cruces, NM 88003, USA; ersieg@nmsu.edu

**Keywords:** hope, psychosocial perceptions, academic achievement, adolescents

## Abstract

This study examined the longitudinal relationships among students’ hope, school-related psychosocial perceptions (i.e., academic self-concept, self-efficacy, academic motivation, and goal valuation), and academic achievement across time via a series of surveys and structural equation models. The study’s sample consisted of 531 ninth grade students from a public high school in a Western state. The study had two primary goals. The first was to examine how students’ hope and school-related psychosocial perceptions predicted their subsequent academic achievement over the course of an academic school year. The second was to explore whether school-related psychosocial perceptions accounted for the positive relationship between students’ hope and their academic achievement that several previous studies have reported. This study had two primary findings. First, both students’ hope and school-related psychosocial perceptions predicted their subsequent academic achievement, with each variable having around a medium effect size. Second, although students’ hope indirectly predicted their subsequent academic achievement via their school-related psychosocial perceptions, academic self-concept and academic motivation were found to be the two most meaningful contributors.

## 1. Introduction

Many scholars over the past three decades have utilized C. R. Snyder’s theory of hope to explore the practical benefits of hopeful thinking (Rand et al., 2020; Snyder et al., 1991). Their discoveries have been wide-ranging, with studies revealing that hopeful thinking is beneficial within areas such as sports performance (Cheavens et al., 2005; Delas et al., 2017), mental health (Marques et al., 2017), cancer recovery (Berendes et al., 2010), and spirituality (Marques et al., 2013). A key theme across this research is that a person’s expectation of a better future positively affects them in the present. One area where this theme is particularly evident is within schools. Several studies that focus on hope within the school context indicate that hopeful thinking meaningfully predicts (a) higher academic motivation (*r* = 0.48, *p* < 0.01, Piña-Watson et al., 2015), (b) higher academic engagement (*r =* 0.52, *p* < 0.001, Van Ryzin et al., 2009), (c) an increased likelihood of engaging in problem-solving behaviors (*r =* 0.35, *p* < 0.01, Atik & Erkan Atik, 2017), and (d) an increased likelihood of making decisions that promote becoming a college graduate (56.52% vs. 40.27%, Snyder et al., 2002).

However, despite the established benefits of hopeful thinking within the school context, there is currently very little known about *how* hopeful thinking may lead to desirable academic outcomes. More specifically, there has been very little empirical research examining the mechanism or process that turns hopeful thinking into better academic outcomes for students. However, recently a prominent theory has been put forth on this very topic. Dixson and colleagues asserted the effective mechanism of hopeful thinking centers around psychosocial perceptions—students’ thoughts, attitudes, and beliefs about themselves within the school context (Dixson et al., 2017b). The theory, called the hope into achievement theory, posits that hopeful thinking leads to more success-oriented psychosocial perceptions, which in turn leads to higher academic achievement (Dixson, 2019a; Dixson et al., 2017b; Dixson & Stevens, 2018). However, despite the numerous studies outlining this framework (e.g., J. Chen et al., 2020; Dixson, 2019a, 2019b; Dixson et al., 2017b; Dixson & Stevens, 2018), as well as the wide-ranging positive implications of this theory (e.g., that hope interventions can widely increase students’ odds of academic success), there has been no research directly examining the empirical feasibility of this theory.

In this study, we examined the longitudinal relationship between students’ hope, school-related psychosocial perceptions, and academic achievement to directly assess whether students’ school-related psychosocial perceptions mediate the relationship between their hope and subsequent academic achievement. We begin with a discussion of hope and school-related psychosocial perceptions, including how they both relate to students’ academic achievement. Next, we discuss both the theoretical and empirical literature surrounding the hope into achievement model. Finally, we report on the present study, which directly tests the model.

## 2. Hope

Hope is defined as a person’s perceived ability to envision a better future, irrespective of their current circumstances, mixed with their confidence to make the envisioned future a reality (Dixson, 2017, 2023; Snyder, 2002). Hope is conceptualized as a cognitive-motivational process that consists of two complementary yet distinct components. The first component is pathways, which refers to one’s perceived ability to envision routes, or pathways, to desired future goals irrespective of one’s current circumstances (Dixson, 2019a; Snyder, 2002). In general, high-pathways thinkers generate more routes to their goals (in preparation of setbacks or impediments), create more elaborate routes to their goals, and are more flexible throughout the goal-achievement process than low-pathways thinkers (Irving et al., 1998; Snyder, 2002). The second component is agency, which refers to one’s perceived ability, motivation, and perseverance to accomplish their future goals via their envisioned routes (Dixson, 2019a; Snyder, 2002). In general, high-agency thinkers display higher work ethic (*r* = 0.40, Dixson, 2019a), motivation (*r* = 0.57, Dixson & Stevens, 2018), and perseverance (*r* = 0.45, Lee & Jang, 2018) throughout the goal-achievement process than low-agency thinkers.

Hope is typically measured with the Children’s Hope Scale (CHS; Snyder et al., 1997) in populations under the age of 18 and with the Adult Hope Scale (AHS; Snyder et al., 1991) in populations aged 18 and above (Dixson, 2017). Several previous studies have outlined how hope is both theoretically and empirically different from the closely related constructs of self-efficacy and optimism (see Rand et al., 2020 for review). For example, Magaletta and Oliver (1999) found that the items of the AHS loaded onto a different factor than the items from commonly used scales of optimism (i.e., the Life Orientation Test; Scheier & Carver, 1985) and self-efficacy (i.e., the Self-Efficacy Scale; Sherer et al., 1982), implying that the items of these scales measure different constructs. In addition, a host of different studies have found that hope shares only a moderate amount of variance with both optimism (10–41%; Feldman & Kubota, 2015; Rand et al., 2011; Shanahan et al., 2020) and self-efficacy (20–47%; Dixson et al., 2016; Feldman & Kubota, 2015; Gallagher & Lopez, 2017), indicating that the majority of its variance is unique (53–90%).

## 3. Hope and Academic Achievement

Several studies have examined the relationship between hope and academic achievement, with the majority indicating that students’ hope scores predict their academic achievement. For example, in a diverse sample of 89 college students, Feldman and Kubota (2015) examined the relationship between hope, self-efficacy, optimism, and academic achievement. They reported two primary findings: (a) of the predictors, hope had the highest correlations with academic achievement (general hope *r* = 0.32, academic hope [i.e., hope within the academic context] *r* = 0.69, optimism *r* = 0.18, self-efficacy *r* = 0.31), and (b) hope was the only significant predictor of academic achievement when all of the constructs were included in a structural equation model (*β* = 0.31). Several other studies mirror these findings. For example, in a series of studies across three different samples, Marques and colleagues found that students’ hope scores meaningfully predicted their academic achievement (total *n* = 1111, *r* range: 0.31–0.36, *p*s < 0.05; Marques et al., 2015, 2011a, 2011b). Similarly, in a different series of studies, Dixson and his colleagues reported an almost identical relationship between hope and academic achievement across four diverse samples (total *n* = 1578, *r* range: 0.24–0.34, *p*s < 0.05; Dixson et al., 2016, 2017a, 2017b).

Previous research also indicates that students’ hope scores predict their academic achievement across time. For instance, in a longitudinal study of the effects of hopeful thinking throughout college, Gallagher et al. (2017) found that students’ (*n* = 229) hope scores in their first semester of college meaningfully predicted their subsequent academic performance (*r* range: 0.36–0.38, *p*s < 0.05) and the number of semesters they were enrolled over the next four years (*r* = 0.28, *p* < 0.01). Moreover, the relationship remained even after controlling for high school grade point average (GPA) and ACT scores (*r* range: 0.30–0.33, *p*s < 0.05). Similarly, J. Chen et al. (2020) found that elementary school students’ hope scores at the beginning of the academic year predicted their subsequent academic achievement throughout the year (three time points, *r* range: 0.17–0.24, *p*s < 0.001). In summary, the empirical literature has consistently found support for a relationship between students’ hopefulness and their academic achievement both concurrently and across time.

## 4. School-Related Psychosocial Perceptions

Psychosocial perceptions within the school context can be defined as students’ thoughts, beliefs, and attitudes about themselves that influence how they behave in school, relate to their school’s context, or value achievement in school (Dixson & Stevens, 2018; Gentzis & Dixson, 2024). These perceptions can be as broad as students’ beliefs about academic achievement or as narrow as a students’ thoughts on their ability to complete a homework assignment. Within this article, four specific psychosocial variables were chosen to broadly represent the four major categories of school-related psychosocial perceptions identified in a seminal meta-analysis on psychosocial perceptions and student achievement (i.e., Robbins et al., 2004).

The first, academic self-concept, is defined as a student’s thoughts and beliefs about their own academic ability (Albert & Dahling, 2016). This variable was chosen to represent students’ academic identities, including how they broadly view themselves within the academic context (e.g., as “a math person”) as well as their more nuanced beliefs about their academic capabilities. Academic self-concept is typically measured using self-report scales (e.g., Academic Self-Concept scale; Reynolds et al., 1980) and has been found to predict influential academic constructs such as educational attainment (*r* = 0.49, *p* < 0.05; Marsh & O’Mara, 2008), school adjustment (*r* = 0.49, *p* < 0.05; Fernández et al., 2012), concentration (*r* = 0.56, *p* < 0.01; Ommundsen et al., 2005), and academic engagement (*r* = 0.51, *p* < 0.01; Dixson, 2019c).

The second, self-efficacy, is defined as a person’s general confidence in their ability to accomplish specific tasks (G. Chen et al., 2001). This variable was chosen to represent students’ beliefs about their ability to complete tasks as well as their perceived initiative and follow-through. Self-efficacy is typically measured using the General Self-Efficacy Scale (Sherer et al., 1982) and has been shown to be influential within the school context via meaningful relationships with work ethic (*r* = 0.34, *p* < 0.01; Dixson, 2019a), academic aspirations (*r* = 0.54, *p* < 0.01; Carroll et al., 2009), and curiosity (*r* = 0.33, *p* < 0.01; Dixson, 2019a).

The third, academic motivation, is defined as a student’s desire to systematically engage in behaviors to produce a valued academic outcome. This variable was chosen to represent a student’s self-regulation and interest in bringing about successful academic outcomes. Academic motivation is typically measured using self-report scales (e.g., the Academic Motivation Scale; Vallerand et al., 1992) and has demonstrated its importance within the school context via meaningful associations with influential constructs such as school enjoyment (*r* = 0.31, *p* < 0.01; Fenzel & O’Brennan, 2007), conscientiousness (*r* = 0.28, *p* < 0.01; Komarraju et al., 2009), students’ feelings about their teachers (*r* = 0.53, *p* < 0.01; Dixson & Stevens, 2018), and students’ use of self-regulation strategies (*r* = 0.75, *p* < 0.01; King & Ganotice, 2014).

The last variable, goal valuation, is defined as a student’s perceived value of positive academic outcomes (McCoach & Siegle, 2003). This variable was chosen to represent students’ academic goals and future orientation, as well as their perceived value of academic outcomes and performance. Goal valuation is most frequently measured using the Goal Valuation subscale of the School Attitude Assessment Survey-Revised scale (McCoach & Siegle, 2003). Previous research indicates that goal valuation is important within the school context, as studies have found that goal valuation meaningfully relates to perceived teacher support (*r* = 0.32, *p* < 0.01; Wang & Eccles, 2013), students’ feelings about their teachers (*r* = 0.75, *p* < 0.01; Erkman et al., 2010), students’ feelings about their school (*r* = 0.76, *p* < 0.01; Erkman et al., 2010), and cognitive engagement in school (*r* = 0.34, *p* < 0.01; Wang & Eccles, 2013).

Altogether, academic self-concept, self-efficacy, academic motivation, and goal valuation represent students’ thoughts about themselves academically, their confidence and motivation to execute tasks, and the personal value they place on desirable academic goals and outcomes. Previous research has emphasized these psychosocial perceptions as the most relevant and consequential regarding students’ academic success (for meta-analyses, see Fong et al., 2017; Robbins et al., 2004).

## 5. Psychosocial Perceptions and Academic Achievement

The research literature on psychosocial perceptions and academic achievement indicates that the two have a positive relationship. For example, Robbins et al. (2004) conducted a comprehensive meta-analysis examining how students’ psychosocial perceptions related to their academic achievement. In their sample of over 100 individual studies (total *n* ≥ 100,000 students), they found that students’ academic motivation (*r* = 0.26) and self-efficacy (*r* = 0.38) significantly predicted their academic achievement. Relatedly, in a different meta-analysis that consisted of almost 200 studies (total *n* ≥ 56,000 students), Fong et al. (2017) reported similar results. They also found that students’ self-perceptions (*r* = 0.21), motivation (*r* = 0.25), and self-regulation (*r* = 0.22) significantly predicted their academic achievement.

Several additional studies indicate a similar pattern of results for the specific psychosocial perceptions focused on within this study. More specifically, several previous studies examining the relationship between students’ academic achievement and their academic self-concept, self-efficacy, academic motivation, and goal valuation indicate that these four psychosocial constructs each have a positive and meaningful relationship with student achievement. In particular, academic self-concept has been reported to have a moderate-to-strong relationship with student achievement (*r* range: 0.60–0.70, *p*s < 0.05; Dixson, 2019c; Dixson et al., 2017b; Guay et al., 2010), and self-efficacy, academic motivation, and goal valuation have been reported to have moderate relationships (self-efficacy, *r* range: 0.30–0.53, *p*s < 0.05; Dixson et al., 2016; Komarraju & Nadler, 2013; Renshaw & Chenier, 2016; academic motivation, *r* range: 0.34–0.42, *p*s < 0.05; Abd-El-Fattah & Patrick, 2011; Bal-Taştan et al., 2018; Dickhäuser et al., 2016; goal valuation, *r* range: 0.29–0.34, *p*s < 0.05; Arslan, 2016; Balkıs et al., 2016; Martin & Steinbeck, 2017). Moreover, a host of longitudinal studies also indicate that academic self-concept (e.g., *r* = 0.51, *p* < 0.05, after a year; Guay et al., 2010), self-efficacy (e.g., *r* = 0.34, *p* < 0.05, after four years; Gallagher et al., 2017), academic motivation (e.g., *r* = 0.39, *p* < 0.05, after a year; Skaalvik & Valås, 1999), and goal valuation (e.g., *r* = 0.41, *p* < 0.05, after a year; Hornstra et al., 2013) independently predict students’ subsequent academic achievement, indicating that these psychosocial perceptions may cause or directly lead to increased academic achievement.

## 6. Hope, School-Related Psychosocial Perceptions, and Academic Achievement

Although several previous studies have (a) linked students’ hope to their subsequent school-related psychosocial perceptions (e.g., Marques et al., 2011b; Van Ryzin et al., 2009), and (b) linked students’ school-related psychosocial perceptions to their subsequent academic achievement (e.g., Skaalvik & Valås, 1999; Walton & Cohen, 2011), as of this writing, no study could be found that links students’ hope to their psychosocial perceptions and then their academic achievement longitudinally. However, there is a prominent theory that may explain how these three constructs are related over time. Dixson et al. (2017b) theorized that students’ hope may indirectly influence their academic achievement via directly influencing the degree to which they adopt a more success-oriented psychosocial profile (i.e., embrace beliefs, attitudes, and perspectives that are conducive to engaging in behaviors that drive academic success, Dixson et al., 2017b). This theory is empirically supported and is consistent with other theories that outline how psychosocial perceptions can improve student achievement over time (e.g., Yeager & Walton, 2011).

This theory is further rooted in empirical research surrounding hope. For example, in a diverse sample of 297 adolescents, Dixson and colleagues (2017) examined how four different hope clusters related to a success-oriented psychosocial profile (Dixson et al., 2017b). The four clusters were *high hopers* (i.e., students who reported high pathways and agency scores), *high agency thinkers* (i.e., students who reported high agency and average to low pathways scores), *high pathways* thinkers (i.e., students who reported high pathways and average to low agency scores), and *low hopers* (i.e., students who reported low pathways and agency scores). Dixson and colleagues found that the high hoper cluster reported a meaningfully more adaptive psychosocial profile than all other clusters. More specifically, the high hoper cluster reported scores that were meaningfully more positive across nine influential school variables (e.g., school belonging, educational expectations, academic investment, academic self-concept, and self-esteem). High-hopers were followed by high agency thinkers, high pathway thinkers, and the low-hopers, respectively, with meaningful separation between the groups (i.e., 50% of the differences across groups had a medium or large effect size). Similarly, in a different study, Dixson and Stevens (2018) examined whether hope would predict a psychosocial profile of achievement in a sample of 117 African American high school students. After controlling for gender, age, parental education, and previous standardized test scores in mathematics and reading, they found that students’ hope scores accounted for 17.2% to 29.9% of their motivation, academic self-concept, school belonging, academic goal valuation, and attitude toward their teachers. These studies provide direct support for the theory that underlies the current study.

Several additional studies provide indirect support for this theory. For instance, Dixson (2019a) conducted a study examining whether hope scores predicted success-oriented behaviors in school, an indirect indicator of both a success-oriented psychosocial profile and student achievement (Dixson & Stevens, 2018). In both a high school (*n* = 447) and college (*n* = 375) sample, he found that students’ hope scores meaningfully predicted their behavioral engagement in class (Cohen’s *d* range: 0.57–1.42), emotional engagement in class (*d* range: 0.49–1.52), and overall work ethic (*d* range: 0.28–1.17), with almost all effect sizes in the medium to large range. Similarly, in a longitudinal study examining the relationship between hope, behavioral engagement, and academic achievement in a sample of 949 elementary school students in China, J. Chen et al. (2020) found that hope not only predicted behavioral engagement (*r* range: 0.36–0.53, *p*s < 0.001) and student achievement (*r* range: 0.17–0.24, *p*s < 0.001) over five time points, but also that behavioral engagement mediated the relationship between hope and academic achievement. Overall, these studies provide foundational empirical support for the hope into achievement theory.

## 7. The Current Study

The current study had two primary goals. The first was to examine how students’ hope and school-related psychosocial perceptions predicted their subsequent academic achievement over the course of an academic school year. The second was to examine whether students’ hope indirectly predicts their subsequent academic achievement via four major categories of school-related psychosocial perceptions (represented within this study by academic self-concept, self-efficacy, academic motivation, and goal valuation). This investigation explored a potential mechanism of how students’ hope may influence their subsequent academic achievement. In addition to being a direct empirical examination of Dixson et al.’s (2017b) hope into achievement theory, the current study will also shed light on how hope and school-related psychosocial perceptions are related to student achievement over time, thus providing additional insight for the development of more informed hope interventions. This examination was guided by three research questions. Consistent with the first primary goal of this study, our first research question was how do students’ hope and school-related psychosocial perceptions relate to their academic achievement over time? Consistent with the second primary goal of this study, our second and third research questions were (a) does students’ hope indirectly predict their subsequent academic achievement via the four major categories of school-related psychosocial perceptions?, and (b) if students’ hope indirectly predicts their subsequent academic achievement via school-related psychosocial perceptions, which categories of school-related psychosocial perceptions best account for the relationship?

In accordance with previous research (e.g., Marques et al., 2011b; Van Ryzin et al., 2009; Walton & Cohen, 2011; Yeager & Walton, 2011), it was hypothesized that (a) students’ hope, academic self-concept, self-efficacy, academic motivation, and goal valuation scores would predict their subsequent GPAs with at least medium effect sizes, and (b) students’ hope scores would indirectly predict their subsequent GPAs via the four major categories of school-related psychosocial perceptions, with all four categories making an equally positive and significant contribution.

## 8. Method

### 8.1. Participants and Procedure

The study sample consisted of 531 ninth-grade adolescents (51.2% female) who attended a diverse public high school of around 3000 students in a Western U.S. state. The self-reported racial/ethnic groups of the participants were 52.4% European American/White, 17.1% Hispanic American/Latinx, 7.5% Asian American/Pacific Islander, 6.6% African American/Black, and 16.0% Multi-Racial/Other. The parental education breakdown of the sample was 4.7% were not high school graduates, 11.5% were high school graduates, 23.0% had some college experience, 55.2% were college graduates, 1.7% had graduate/post-graduate degrees, and 3.8% declined to state or were unknown. Parental education level was obtained from the school records of the sample’s participants.

The current study was approved by the Institutional Review Boards (IRBs) in the school district and at the first author’s university. Data were collected via two school-administered surveys throughout the 2018–2019 academic year. The goal of these surveys was to better understand student perceptions about the school climate so the school administration could better address student needs. Time 1 data were collected at the onset of the 2018 Fall Semester and Time 2 data were collected within the first four weeks of the 2019 Spring Semester. Student GPAs were obtained at the end of the 2019 Spring Semester (Time 3) from school records. For both waves of survey data collection, about 800 students were invited to participate in the current research. A response rates of 78.25% (*n* = 626) and 73.30% were obtained for the first and second waves of data collection, respectively. Students had to complete both surveys in order to be included in the current research. The current study had an 84.82% (531 of 626) retention rate (i.e., the percentage of students who completed the second survey after completing the first). It should be noted that students were allowed to take the second survey whether or not they completed the first. Given that the current study had below 20% attrition, it was considered at low risk for bias (Babic et al., 2019).

Prior to the survey, students were notified of their right to decline participation. Students who did not provide assent were excluded from the study. Each survey administration occurred during a pre-arranged school-wide free period during the school day. Students accessed the surveys via an anonymous link from their teacher. While completing the survey, students provided their student identification (ID) number so that responses across all time points could be linked together. After all student responses were linked, student ID numbers were purged from the dataset. All students completed the surveys in a single session and it generally took them about 20 to 30 minutes to complete each survey. If students had questions during any administration, teachers were available to answer them. Given that teachers were not trained, they only answered rudimentary questions such as those centered around word definitions and the purpose of the survey. Students did not receive any compensation for their participation in this study.

### 8.2. Measures

#### 8.2.1. Hope

Hope was measured using the Children’s Hope Scale (CHS; Snyder et al., 1997). The CHS is composed of six items that measure hope via two complementary subscales—pathways and agency. Three items of the CHS measure pathways (e.g., “When I have a problem, I can come up with lots of ways to solve it”) and three items measure agency (e.g., “I think I am doing pretty well”). Pathways and agency scores were combined to create a total hope score, and all items were rated on a 5-point Likert scale ranging from 1 (*Strongly disagree*) to 5 (*Strongly agree*). Previous research supports the theory-consistent 2-factor structure of the CHS (see Dixson, 2017; and Valle et al., 2004) and scores on the CHS have been found to be internally consistent, with alpha estimates ranging from 0.70 to 0.85 (Dixson, 2017; Valle et al., 2004). In the current sample, scores on the CHS were concluded to be reliable and structurally valid with a 2-factor confirmatory factor analysis (CFA) yielding acceptable fit indices (i.e., CFI ≥ 0.90; TLI ≥ 0.90; Marsh et al., 2004) and factor loadings (i.e., >0.40; Watkins, 2018), in addition to an alpha estimate above the commonly accepted cutoff of 0.70 (see Table 1 and Table 2).

#### 8.2.2. Academic Self-Concept

Academic self-concept (ASC) was assessed using four items that measured students’ perceptions about their own academic ability. The ASC items were “I have the ability to get an A in Math,” “I have the ability to get an A in English,” “I have the ability to get an A in Physics,” and “I have the ability to get an A in Ethnic Studies.” These specific subjects were chosen because all ninth-grade students were required to be enrolled in mathematics, English, physics, and ethnic studies throughout the entire year of the study. Response options were a 5-point Likert scale ranging from 1 (*Strongly disagree*) to 5 (*Strongly agree*), with higher scores indicating higher perceived academic ability. Similar ASC scales have been used in other studies to measure ASC effectively (e.g., Dixson, 2019c). Scores on the included ASC scale were concluded to be structurally valid and reliable in the current sample with an alpha estimate confidence interval that fell within the range of acceptability as well as factor loadings and fit indices (1-factor) that were above acceptable cutoffs (see Table 1 and Table 2). Further, correlations within the current sample between ASC and the other included variables (e.g., motivation and student GPAs) were meaningful and consistent with theory (e.g., Guay et al., 2010), providing some evidence of convergent validity.

#### 8.2.3. Self-Efficacy

Self-efficacy was measured using the New General Self-Efficacy Scale (NGSE; G. Chen et al., 2001). The NGSE scale consisted of eight items that measured students’ perceived ability to accomplish specific tasks (e.g., “I am confident that I can perform effectively on many different tasks”). Response options were on a 5-point Likert scale ranging from 1 (*Strongly disagree*) to 5 (*Strongly agree*), with higher scores indicating a higher perceived capacity to accomplish a certain task. Evidence from previous research indicates that NGSE scale scores are structurally sound and best fit a 1-factor structure (see G. Chen et al., 2001). In addition, scores on the NGSE scale have been found to be internally consistent, with previous alpha estimates ranging from 0.84 to 0.86 (see Azizli et al., 2015; G. Chen et al., 2001). In the current sample, scores on the NGSE scale were found to be both structurally sound and reliable, with fit indices, factor loadings, and alpha estimates all surpassing commonly accepted cutoffs (see Table 1 and Table 2).

#### 8.2.4. Academic Motivation

Academic motivation was measured using the Motivation/Self-regulation subscale (MSR) of the School Attitude Assessment Survey-Revised (SAAS-R; McCoach & Siegle, 2003). The MSR consisted of 10 items that measured students’ academic motivation (e.g., “I am motivated to do my schoolwork”). Response options were on a 5-point Likert scale ranging from 1 (*Strongly disagree*) to 5 (*Strongly agree*), with higher scores indicating higher academic motivation. Scores on the MSR have previously been found to be internally consistent (alpha range: 0.87–0.94; McCoach & Siegle, 2003; Suldo et al., 2007) and structurally sound (1-factor solution with acceptable factor loadings and fit indices; see Dixson & Stevens, 2018; McCoach & Siegle, 2003). In the current sample, MSR scores were concluded to be structurally sound and reliable, with factor loadings, fit indices, and alpha estimates all above commonly accepted cutoffs (see Table 1 and Table 2).

#### 8.2.5. Goal Valuation

Goal valuation was measured using the Goal Valuation (GV) subscale of the SAAS-R (McCoach & Siegle, 2003). The GV subscale consisted of six items that measured the perceived value of academic goals to students (e.g., “It’s important to get good grades in school”). Response options were on a 5-point Likert scale ranging from 1 (*Strongly disagree*) to 5 (*Strongly agree*), with higher scores indicating a higher perceived value and utility of academic goals. Scores on the GV subscale have been found internally consistent (e.g., α = 0.89) and structurally sound, with the factor loadings of previous exploratory factor analyses (e.g., range: 0.56–0.91) being found acceptable (Dixson & Stevens, 2018; McCoach & Siegle, 2003). Similarly, in the current sample, GV scale scores were also concluded to be reliable and structurally sound with an alpha estimate, factor loadings, and fit indices all within acceptable range (see Table 1 and Table 2).

#### 8.2.6. Grade Point Average

Academic achievement was measured via students’ 2018–2019 cumulative GPA. GPAs were obtained from school records and were reported on a 4-point weighted scale (with A = 4.0, B = 3.0, C = 2.0, D = 1.0, and F = 0.0). Students’ GPAs were calculated by averaging their academic performance within each class across both semesters, then averaging their performance across classes. Although pluses and minuses were reported on students’ report cards, they did not affect their GPAs; thus a B+, B, and B− all contributed equally to a student’s GPA (i.e., 3.0).

## 9. Results

### 9.1. Preliminary Analyses

Descriptive statistics including means, standard deviations, and intercorrelations of study variables are presented in Table 1. Gender and racial differences across study variables were examined using a series of t-tests and ANOVAs, respectively. Several statistically significant differences were found in keeping with the existing literature (e.g., Dixson et al., 2017b). Within the current sample, European American and Asian American students reported higher goal valuation than Hispanic students (European American, Hedge’s *g* = 0.37, *p* = 0.015; Asian American, *g* = 0.46, *p* = 0.044) and higher GPAs than both African American (European American, *g* = 1.08, *p* < 0.001; Asian American, *g* = 0.81, *p* = 0.003) and Hispanic students (European American, *g* = 0.93, *p* < 0.001; Asian American, *g* = 0.68, *p* < 0.001). European American students also reported higher academic self-concept scores than Hispanic students (*g* = 0.55, *p* < 0.001). Females reported higher goal valuation (*g* = 0.41, *p* < 0.001) and GPAs (*g* = 0.27, *p* = 0.002) than males, while males reported higher hope (*g* = 0.28, *p* < 0.001). All other differences across study variables were non-significant (i.e., *p*s > 0.05). Consistent with best practice (Schlomer et al., 2010), missing data were imputed using the Expectation-Maximization algorithm (10 iterations). Prior to imputation, data were found to be missing completely at random (Little MCAR test = *p*s > 0.05); 0.0% to 1.9% of data were imputed per item that made up the psychosocial perceptions (no student GPAs were imputed).

### 9.2. How Hope and School-Related Psychosocial Perceptions Relate to Academic Achievement Over Time

A series of correlations were conducted to examine the relationships between hope, academic self-concept, self-efficacy, academic motivation, and goal valuation to student academic achievement over time. As can be seen in Table 1, Time 1 hope scores were meaningfully related to student GPAs about nine months later, with an effect size approaching medium. This indicates the more hopeful a student was at the beginning of the school year, the better they performed academically throughout the school year. In addition, academic self-concept, self-efficacy, academic motivation, and goal valuation scores at Time 2 also predicted student GPAs about four months later, all with medium effect sizes (see Table 1). This indicates that as students (a) believed in their academic abilities more, (b) believed in their ability to accomplish the tasks that they set their minds to more, (c) were more motivated within the school context, and (d) valued academic outcomes more at the beginning of the second semester, the better they performed academically over the second half of the school year.

### 9.3. Hope into Achievement Theory Model

A series of structural equation models (SEM) were conducted to examine the hope into achievement theory model—whether hope indirectly predicts student achievement via the four major areas of school-related psychosocial perceptions. Consistent with best practice, all SEMs were carried out using the weighted least squares means and variance adjusted (WLSMV) estimator (as five of six study variables were ordinal; Muthén & Muthén, 2014) and model acceptability was judged based on factor loadings, alpha estimates, multiple fit indices, and previous theory. For factor loadings, consistent with previous research (Watkins, 2018), factor loadings above 0.40 were considered acceptable. With regards to fit indices, the Tucker Lewis Index (TLI), Comparative Fit Index (CFI), and Root Mean Square Error of Approximation (RMSEA) were interpreted. Consistent with previous research (Kenny, 2024; Marsh et al., 2004), *acceptable fit* was indicated by TLI and CFI values of 0.90–0.95 and RMSEA values within the range of 0.05–0.08. *Close* or *good fit* was indicated by TLI and CFI values above 0.95 and RMSEA values below 0.05 (Kenny, 2024; Marsh et al., 2004). It is important to note that RMSEA values were not interpreted for the measurement models within this study as previous research indicates they are artificially high in models with low degrees of freedom (Kenny, 2024; Kenny et al., 2014). The chi square was not interpreted at all in the current study as previous research indicates that it is statistically significant in most sample sizes above 200 (Kenny, 2024).

**Figure 1:**
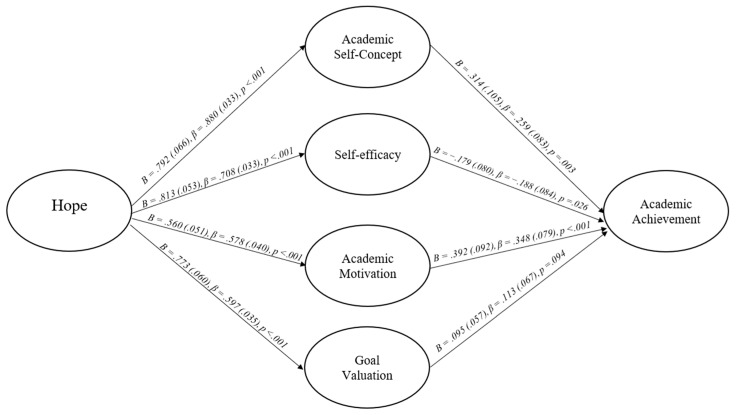
Hope into achievement theory model.

The factor loadings, fit indices, indirect paths, and path models of the hope into achievement model are presented in Figure 1 and Table 2 and Table 3. The hope into achievement theory model starts with hope, simultaneously passes through academic self-concept, self-efficacy, academic motivation, and goal valuation, and ends with cumulative student GPA. Consistent with previous studies indicating that psychosocial factors are related (McCoach & Siegle, 2003; Pajares & Graham, 1999), all four psychosocial constructs collected at Time 2 were fixed to correlated within the model (*r* range: 0.07 [academic motivation and academic self-concept]–0.21 [academic motivation and goal valuation]). As can be seen in Table 2, the hope into achievement model exhibited acceptable factor loadings with the factor loadings of all latent constructs being greater than 0.52, as well as a CFI, TLI, and RMSEA statistic that fell within the acceptable range. Overall, the model accounted for 25.2% of GPA, 77.4% of academic self-concept, 50.1% of self-efficacy, 33.5% of academic motivation, and 35.6% of goal valuation. As a result of the acceptable factor loadings, fit indices, alpha estimates, and the model being consistent with previous theory (Dixson et al., 2017b), the model was concluded to be interpretable.

All paths, both direct and indirect, within the model are presented in Figure 1 and Table 3. As can be seen, hope significantly predicted all four school-related psychosocial perceptions with *B*s and standardized betas within the moderate to large range. Academic self-concept, self-efficacy, and academic motivation then significantly predicted students’ cumulative GPAs with *B*s and standardized betas within the small to moderate range. The indirect paths mirrored the direct paths. The total indirect path from hope to students’ cumulative GPAs was positive, statistically significant, and had a moderately sized *B* and *β*. The paths from hope to student GPA via academic self-concept and academic motivation were also both positive and statistically significant. The path via self-efficacy was negative and statistically significant, while the path via goal valuation was not statistically significant.

**Figure 2:**
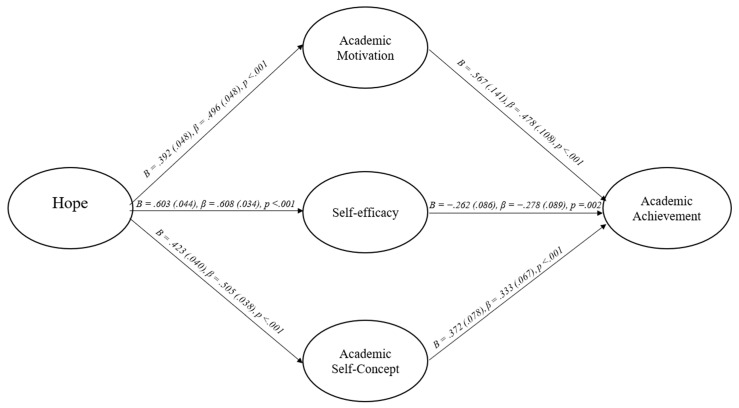
Hope into achievement theory model (without goal valuation, Model 2).

Given that one of the goals of the current manuscript was to better understand the mechanism of how students’ hope may influence their subsequent academic achievement, two additional SEMs were conducted to explore more optimal theoretical models in light of the first model results. The first additional model explored was identical to the hope into achievement theory model, except goal valuation was removed as it did not significantly predict students’ academic achievement in the initial model. This second model (Model 2) exhibited acceptable factor loadings (i.e., all ≥0.56) and all fit indices were within the acceptable range (see Table 2 and Table 3). It was also consistent with previous theory (Dixson et al., 2017b). As a result, it was concluded to be interpretable. The results of the SEM (see Figure 2) consisted of hope significantly predicting academic self-concept, self-efficacy, and academic motivation, with positive *B*s and standardized betas within the moderate to large range. Then, academic self-concept and academic motivation significantly predicted student’s academic achievement with positive *B*s and standardized betas within the moderate range, while self-efficacy significantly predicted student’s academic achievement with a negative *B* and standardized beta within the small to moderate range. The indirect paths followed a similar pattern. The path from hope to students’ GPAs was positive and significant for academic self-concept, academic motivation, and for the model as a whole, while it was significant and negative for self-efficacy. All *B*s and standardized betas were in the small to moderate range. The model explained 29.3% of GPA, 24.6% of academic self-concept, 36.9% of self-efficacy, and 25.5% of academic motivation. The correlation among the Time 2 psychosocial constructs ranged from 0.17 to 0.24.

**Figure 3:**
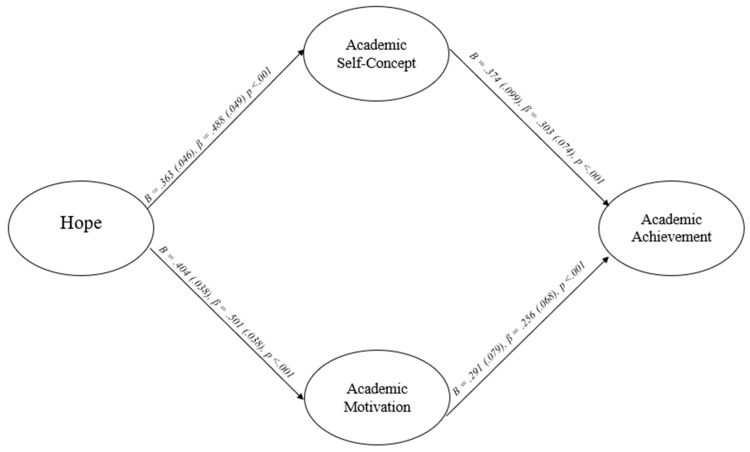
Hope into achievement theory model (without goal valuation and self-efficacy, Model 3).

Given that it is counter to theory that the model would consist of a negative path from self-efficacy to academic achievement, a supplemental model was conducted to examine the possibility of suppression effects. In this supplemental model, after academic motivation was removed, the paths from hope to academic achievement via self-efficacy and academic self-concept were statistically significant and positive, with moderate *B*s and standardized betas. This indicates that academic motivation suppressed the path from hope to academic achievement via self-efficacy in the current model and caused the path from self-efficacy to academic achievement to be negative instead of positive. As a result, a third model was conducted to remove this suppression effect. The third and final model (Model 3) was also identical to the hope into achievement theory model except it only included the school-related psychosocial factors of academic self-concept and academic motivation—the two constructs that were statistically significant positive predictors in the results of the previous two models. This model (Model 3) exhibited acceptable factor loadings (i.e., all ≥0.52), two fit indices within the acceptable range, one fit indices within the good range, and is consistent with previous theory (see Table 2 and Table 3; Dixson et al., 2017b). This model was concluded to be interpretable. The results of the SEM (see Figure 3) consisted of hope significantly predicting both academic self-concept and academic motivation with positive *B*s and standardized betas within the moderate range. Then, both academic self-concept and academic motivation were significant and positive predictors of students’ academic achievement, with positive *B*s and standardized betas within the small to moderate range. The indirect paths, including the path via academic self-concept, via academic motivation, and overall, were all positive and statistically significant with positive *B*s and standardized betas within the small to moderate range. This model accounted for 26.6% of GPA, 23.9% of academic self-concept, and 25.1% of academic motivation. The correlation between academic self-concept and academic motivation was 0.60.

## 10. Discussion

The current study had two goals. The first was to examine the longitudinal relationship between students’ hope and school-related psychosocial perceptions to their subsequent academic achievement. The second goal was to better understand the potential mechanism of how students’ hope may influence their subsequent academic achievement via examining a promising theory (i.e., Dixson et al., 2017b). More specifically, this study examined whether students’ hope scores were related to their academic achievement via four major categories of school-related psychosocial perceptions (represented within this manuscript by academic self-concept, self-efficacy, academic motivation, and goal valuation). Consistent with these goals, the current study had three hypotheses.

First, it was hypothesized that students’ hope and school-related psychosocial perceptions would meaningfully relate to their academic achievement over time with at least a medium effect size. This hypothesis was mostly supported. Students’ hope, academic self-concept, self-efficacy, academic motivation, and goal valuation scores did significantly predict their subsequent cumulative GPAs, but only the school-related psychosocial perceptions had medium effect sizes (the effects size for hope was approaching a medium effect size). This finding is both consistent with and expands several previous studies that reported similar relationships but could not determine directionality or provide any evidence to suggest a causal relationship due to their cross-sectional methodology (e.g., Dixson et al., 2017a; Marques et al., 2017; Robbins et al., 2004). The current study supports the assertion that increasing students’ hope and school-related psychosocial factors *can* lead to higher subsequent academic achievement. It is important to note that the former *does not* guarantee the latter, but these findings indicate directionality and provide evidence of the possibility.

Second, it was hypothesized that students’ hope would indirectly predict their academic achievement via the four major categories of school-related psychosocial perceptions. This hypothesis was partially supported. The hope into achievement theory SEM (Model 1) was concluded to be interpretable. Results indicated that three of the four indirect paths from students’ hope to their subsequent academic achievement were statistically significant, with the paths via academic self-concept and academic motivation being positive and moderate, while the path via self-efficacy was negative with a small relative effect size. This model accounted for about a fourth of students’ academic achievement at the end of the school year (25.2%). However, although this model provides direct support for Dixson et al.’s (2017b) theory that students’ hope influences their subsequent academic achievement via their psychosocial perceptions, it also indicated that some types of school-related psychosocial perceptions are more important than others (Dixson et al., 2017b).

Third, it was hypothesized that all four major categories of school-related psychosocial perceptions would equally account for the positive relationship between students’ hope scores and their subsequent academic achievement. This was also partially supported as only three of the four indirect paths within the hope into achievement model were statistically significant. To further clarify which categories best accounted for the relationship between students’ hope and their subsequent academic achievement (i.e., which categories of school-related psychosocial perceptions were potential mechanisms), two additional models were examined. Model 2 was the initial model without goal valuation, to remove the non-significant indirect path. Although Model 2 was also found to be interpretable, it contained a statistically significant negative path from students’ hope scores to their subsequent academic achievement via self-efficacy. This path was found to be the result of model suppression. As a result, Model 3 was conducted to remove the suppressed path from the model as well. Model 3 was also found to be interpretable, with two positive, statistically significant paths from students’ hope scores to their subsequent academic achievement via academic self-concept and academic motivation. Moreover, this simpler model accounted for a similar amount of students’ academic achievement as the previous models (25.1% for Model 3 vs. 25.2% for Model 1 and 29.3% for Model 2). Altogether, these results indicate that school-related psychosocial perceptions may be an influential mechanism through which students’ hope positively relates to their subsequent academic achievement, with academic self-concept and academic motivation being the two categories that best account for the relationship.

Students’ hope indirectly influencing their subsequent academic achievement via academic self-concept and academic motivation is a framework that is consistent with previous research that indicates increased hopeful thinking is associated with more success-oriented thoughts and actions (J. Chen et al., 2020; Dixson, 2019a; Dixson & Stevens, 2018; Dixson et al., 2017b; Feldman & Kubota, 2015; Van Ryzin et al., 2009). Moreover, the findings from Model 3 also provide direct empirical support for Dixson and colleagues’ theory that hopeful thinking increases student achievement by leading students to have more success-oriented psychosocial perceptions. More specifically, this study indicates that an increase in hopeful thinking can precede an increase in more favorable academic beliefs about the self as well as higher academic motivation, which can then precede higher academic achievement. There are several potential wide-ranging implications of this study’s results.

### 10.1. Study Implications

First, this study indicates that students’ psychosocial perceptions matter within the school context and they should be concertedly cultivated. This study found that five psychosocial perceptions (i.e., hope and the school-related psychosocial perceptions) meaningfully predicted subsequent student achievement as well as altogether accounted for a quarter of students’ academic achievement. Cultivating higher academic achievement is almost universally prioritized within school boards and among school administrators (Bottoms & Schmidt-Davis, 2010). However, initiatives typically aim to increase students’ academic achievement via direct academic supports (e.g., small group learning, tutoring, and increased instruction time; Bottoms & Schmidt-Davis, 2010). This study’s results indicate that school administrations should also consider targeting students’ maladaptive (i.e., low or negative) psychosocial perceptions as that might help to more comprehensively aid the improvement of their academic achievement. This point is bolstered by Hattie (2018) who, in a meta-analysis of meta-analyses, found that tutoring (*d* = 0.26), homework (*d* = 0.29), and increased instruction time (i.e., summer school, *d* = 0.23) all had lower relationships with achievement than character programs (*d* = 0.34), student motivation (*d* = 0.42), student effort (*d* = 0.77), and student efficacy beliefs (*d* = 0.92). Taken together, prior research and the current study’s findings indicate that interventions focusing on influential psychosocial perceptions could lead to meaningful positive change in students’ subsequent academic achievement and should be considered seriously by stakeholders in education.

A second implication of this study is that it provides insight into how psychosocial perceptions relate to one another within the school context. More specifically, this study’s results indicate that psychosocial perceptions can have a hierarchical relationship with one another, with some psychosocial perceptions (e.g., hope) facilitating multiple other psychosocial perceptions (e.g., academic self-concept and academic motivation). Relatedly, similar to previous research (e.g., Dixson et al., 2024), this study also indicates that all psychosocial perceptions are not created equally within the school setting. Within the current research, goal valuation and self-efficacy were not found to be as closely tied to academic achievement as academic self-concept and academic motivation. Although this could be the result of those psychosocial perceptions not being as directly related to the tasks that students are graded for as academic self-concept and academic motivation (e.g., homework assignments, class participation), it is still important to highlight that some psychosocial perceptions may generate a higher return on investment than others within the school setting. Altogether, these findings can help both school personnel and researchers understand that targeting specific influential psychosocial perceptions (e.g., hope) within the school context can have a positive cascading impact on a student’s psychosocial academic profile. Prior to this study, psychosocial interventions targeted single psychosocial perceptions with the goal of only facilitating the targeted psychosocial construct (e.g., school belonging interventions to engender feelings of school belonging; Walton & Cohen, 2011). Now, researchers should be encouraged to target a single psychosocial construct with the goal of facilitating a success-oriented psychosocial profile. This assertion is supported by other research that indicates that targeting specific psychosocial perceptions within the school context can change a student’s psychosocial trajectory toward higher academic achievement (Yeager & Walton, 2011).

A third and final implication of the current research is the potential of universal hope interventions in schools. Hope interventions have previously been found to be quick (i.e., <90 min; Feldman & Dreher, 2012), effective (i.e., average *d* = 0.40 for single session; Weis & Speridakos, 2011), and have long-lasting effects (i.e., *d* = 0.50 at 18-month follow-up; Marques et al., 2011a). In addition, J. Chen et al. (2020) found that students’ academic achievement predicts their subsequent hope (*r* = 0.26, *p* < 0.001). These findings combined with the third hope into achievement theory model (Model 3) indicate that increasing the hope of students via intervention could begin a positive feedback loop. The theoretical feedback loop would consist of (a) increased hope after a 90-min universal intervention, (b) increased hope promoting increased academic self-concept and academic motivation, (c) increased academic self-concept and academic motivation facilitating increased academic achievement, and (d) increased academic achievement promoting increased hope. Such a chain reaction would be consistent with both seminal theories and studies previously established in the psychosocial literature (i.e., Walton & Cohen, 2011; Yeager & Walton, 2011).

Altogether, this study indicates that a student’s hope, and their psychosocial perceptions more broadly, have ramifications for their subsequent academic achievement. Put simply, this study indicates that hope matters within the school context. Getting students to believe that they can accomplish their goals is a cause that should be actively cultivated by school personnel and researched further by scholars in the future. It should be noted that this study does not indicate that hope is a silver bullet perception that, if leveraged, will overcome the multitude of other factors that contribute to a student’s academic achievement (e.g., poverty and racism). However, the results do indicate that leveraging hope as a part of a larger strategy to improve students’ academic achievement is likely to be beneficial in facilitating students’ academic success.

### 10.2. Limitations

This study, like all research, had several limitations. First, this study was not a psychological experiment. As a result, causal inferences cannot be drawn from the current study’s data. Thus, to both generalize and determine causality, future hope research should replicate the current study’s findings using the methodology of a psychological experiment with proper controls. Second, the current study’s sample was not very geographically, ethnically, or socioeconomically diverse. The data for the current study all came from a single high school in a Western U.S. state, was disproportionately European American, and consisted mostly of students that had college-educated parents. Any of these variables (i.e., geographical, cultural, or socioeconomic) could have changed the interpretation or moderated the results. Future research should replicate the current study’s findings with a more diverse sample to examine whether they generalize to more diverse populations. This is particularly important in light of recent research that indicates that hope is a key variable for certain minoritized populations (e.g., African Americans; Dixson & Gentzis, 2023). Third, with the exception of GPA, all of the current study’s data was collected via school-administered surveys. As a result, the relationships among study variables may be inflated due to common method variance. Consistent with best practice, future research should seek to examine the relationships among hope, academic self-concept, self-efficacy, academic motivation, goal valuation, and academic achievement using different methodologies across various respondents (e.g., student, parent, and teacher). Doing so would provide a more comprehensive picture of how these constructs relate to one another within the school context.

## 11. Conclusions

The current study moves the hope literature forward in meaningful ways. It provides a theoretical framework to better understand hope as an influential construct within the school setting as well as provides some insight into how hope potentially brings about desirable academic outcomes like increased academic achievement. At a minimum, given hope’s potential to be leveraged as a universal hope intervention in schools, this study gives students, teachers, school administrators, and scholars a little hope for a better tomorrow.

## Figures and Tables

**Table 1:** Descriptive Statistics and Correlations for Study Variables.

	1	2	3	4	5	6	*M*	*SD*	α (95% CI)
1. Hope_T1	–						3.74	0.67	0.81 (0.79–0.84)
2. Academic Self-Concept_T2	0.39	–					4.27	0.67	0.66 (0.61–0.71)
3. Self-Eficacy_T2	0.51	0.65	–				3.79	0.64	0.88 (0.86–0.90)
4. Academic Motivation_T2	0.43	0.54	0.62	–			3.69	0.67	0.86 (0.84–0.88)
5. Goal Valuation_T2	0.26	0.59	0.53	0.57	–		4.43	0.62	0.90 (0.88–0.91)
6. Grade Point Average_T3	0.25	0.47	0.33	0.45	0.40	–	3.45	0.72	0.90 (0.88–0.91)

All *p*s < 0.001.

**Table 2:** Factor Loadings and Fit Indices for Study Models Derived from Structure Equation Modeling (WLSMV).

Model	*χ* ^2^	df	CFI	TLI	RMSEA	(90% C.I.)	Factor Loadings
Measurement Models							
Hope_T1	32.53 *	9	0.988	0.979	0.070	(0.045, 0.097)	0.56–0.83
Academic Self-Concept_T2	16.84 *	2	0.984	0.951	0.118	(0.071, 0.173)	0.50–0.80
Self-Efficacy_T2	135.35 *	20	0.976	0.967	0.104	(0.088, 0.121)	0.64–0.80
Academic Motivation_T2	173.40 *	27	0.970	0.960	0.101	(0.087, 0.116)	0.49–0.81
Goal Valuation_T2	43.50 *	9	0.993	0.989	0.085	(0.061, 0.111)	0.80–0.89
Theorized Hope into Achievement Theory Model (Model 1)							
Hope-Psychosocial Perceptions-Achievement	1978.84 *	515	0.915	0.907	0.073	(0.070, 0.077)	
Hope_T1							0.52–0.67
Academic Self-Concept_T2							0.60–0.79
Self-Efficacy_T2							0.66–0.80
Academic Motivation_T2							0.54–0.80
Goal Valuation_T2							0.79–0.90
Theorized Hope into Achievement Theory Model (Minus Goal Valuation; Model 2)							
Hope-Psychosocial Perceptions-Achievement	1065.79 *	342	0.943	0.937	0.063	(0.059, 0.067)	
Hope_T1							0.61–0.78
Academic Self-Concept_T2							0.61–0.78
Self-Efficacy_T2							0.66–0.80
Academic Motivation_T2							0.56–0.81
Theorized Hope into Achievement Theory Model (Minus Goal Valuation and Self-Efficacy, Model 3)							
Hope-Psychosocial Perceptions-Achievement	528.64 *	166	0.952	0.945	0.064	(0.058, 0.070)	
Hope_T1							0.59–0.79
Academic Self-Concept_T2							0.59–0.79
Academic Motivation_T2							0.52–0.79

* *p* values < 0.001.

**Table 3:** Indirect Paths of Study Models Derived from Structure Equation Modeling (WLSMV).

Path	*b*	*S.E.*	*β*	*S.E.*	*p*
Hope into Achievement Theory Model (Model 1)					
Total Indirect	0.396	0.047	0.364	0.036	<0.001
Hope_T1—Academic Self-Concept_T2—GPA_T3	0.249	0.082	0.228	0.075	0.002
Hope_T1—Self-efficacy_T2—GPA_T3	−0.145	0.065	−0.133	0.059	0.024
Hope_T1—Academic Motivation_T2—GPA_T3	0.220	0.056	0.201	0.050	<0.001
Hope_T1— Goal Valuation_T2—GPA_T3	0.073	0.045	0.067	0.041	0.098
Hope into Achievement Theory Model (Without Goal Valuation, Model 2)					
Total Indirect	0.222	0.034	0.236	0.033	<0.001
Hope_T1—Academic Self-Concept_T2—GPA_T3	0.222	0.060	0.237	0.062	<0.001
Hope_T1—Self-efficacy_T2—GPA_T3	−0.158	0.053	−0.169	0.055	0.003
Hope_T1—Academic Motivation_T2—GPA_T3	0.157	0.037	0.168	0.038	<0.001
Hope into Achievement Theory Model (Without Goal Valuation and Self-Efficacy, Model 3)					
Total Indirect	0.254	0.031	0.276	0.029	<0.001
Hope_T1—Academic Self-Concept_T2—GPA_T3	0.136	0.039	0.148	0.041	<0.001
Hope_T1—Academic Motivation_T2—GPA_T3	0.118	0.034	0.128	0.036	<0.001

## Data Availability

The data presented in this study are restricted because of a data-sharing agreement. Depending on the request, they may be available upon request from the corresponding author.
